# Mitotic granule cell precursors undergo highly dynamic morphological transitions throughout the external germinal layer of the chick cerebellum

**DOI:** 10.1038/s41598-019-51532-y

**Published:** 2019-10-23

**Authors:** Michalina Hanzel, Victoria Rook, Richard J. T. Wingate

**Affiliations:** 1 MRC Centre for Neurodevelopmental Disorders, King’s College London, Institute of Psychiatry, Psychology and Neuroscience, 4th floor New Hunt’s House, Guy’s Campus, London, UK; 2 School of Biological and Chemical Sciences, Queen Mary University of London, Mile End Road, London, E4 1NS UK

**Keywords:** Cell fate and cell lineage, Neural progenitors

## Abstract

The developing cerebellum of amniotes is characterised by a unique, transient, secondary proliferation zone: the external germinal layer (EGL). The EGL is comprised solely of granule cell precursors, whose progeny migrate inwardly to form the internal granule cell layer. While a range of cell morphologies in the EGL has long been known, how they reflect the cells’ differentiation status has previously only been inferred. Observations have suggested a deterministic maturation from outer to inner EGL that we wished to test experimentally. To do this, we electroporated granule cell precursors in chick with plasmids encoding fluorescent proteins and probed labelled cells with markers of both proliferation (phosphohistone H3) and differentiation (Axonin1/TAG1 and NeuroD1). We show that granule cell precursors can display a range of complex forms throughout the EGL while mitotically active. Overexpression of full length NeuroD1 within granule cell precursors does not abolish proliferation, but biases granule cells towards precocious differentiation, alters their migration path and results in a smaller and less foliated cerebellum. Our results show that granule cells show a greater flexibility in differentiation than previously assumed. We speculate that this allows the EGL to regulate its proliferative activity in response to overall patterns of cerebellar growth.

## Introduction

Transit amplification of basal progenitors is an important feature of the vertebrate brain development that allows for a rapid expansion of specific cell populations and has facilitated the evolution of foliated structures such as the cortex and the cerebellum^1–3^. Secondary proliferation may also allow dedicated progenitors to respond to local environmental conditions to populate neural structures as required during development and repair. The process is found, for example, in the progenitors in the subventricular zone that generate the migrating neuroblasts of the rostral migratory stream (RMS)^4^, the basal neocortical progenitors^1,5,6^, and granule cell progenitors in the external germinal layer (EGL) of the cerebellum^7–17^.

**Figure 1:**
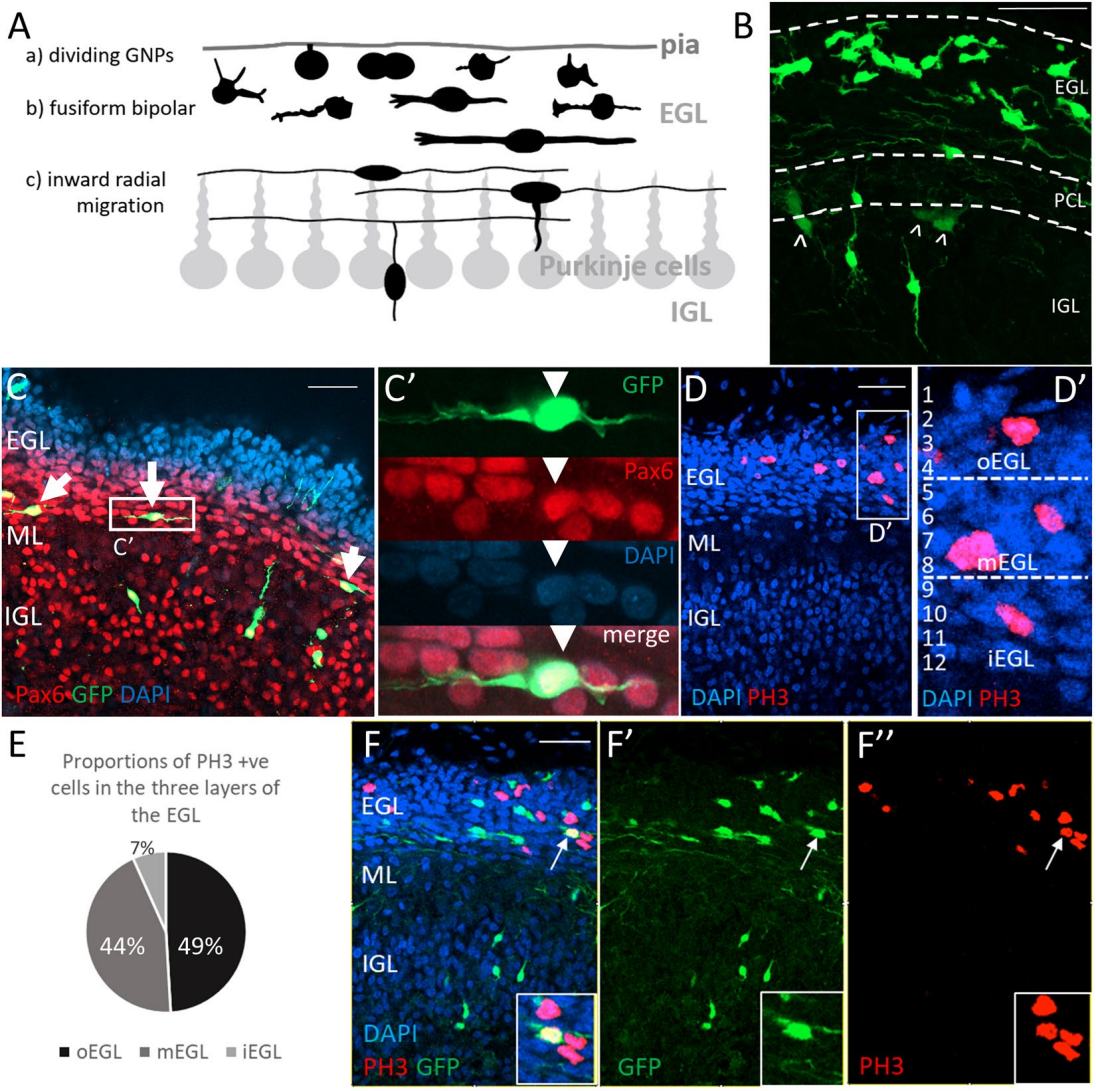
Following E4 rhombic lip electroporation granule cells at various stages of differentiation populate the cerebellar cortex and proliferate throughout the EGL at E14. (**A**) Cajal divided granule cell development in the EGL into three phases: (a) a proliferative phase in superficial EGL (although Cajal did not draw mitotic profiles per se); (b) a bipolar phase with tangential migration; (c) an inward somatic migration to the IGL, through the Purkinje cell layer. Adapted from the Golgi staining drawings from Cajal, 1911^18^. (**B**) An example of E14 chick cerebellar slice following an E4 electroporation at the rhombic lip with Tol2:GFP. The GFP-expressing cells resemble granule cells described by Cajal and others. A small number of cells with other morphologies are also found outside of the EGL and could represent unipolar brush cells (^), born at the rhombic lip after GCPs and residing in the IGL in the adult cerebellum. (**C**) All GFP positive cells found in the inner EGL at E14 co-express the definitive granule cell marker Pax6 (arrows), confirming their identity as granule cells. (**C’**) An example of an electroporated bipolar cell located in the inner EGL co-expressing Pax6. (**D**) EGL of E14 embryos was divided into three equal layers (**D’**) and the numbers of PH3 positive cells in each layer was quantified. (**E**) PH3 positive cells are found throughout the EGL (n = 1345 cells in 15 slices of 3 cerebella). The highest proportion of mitotic cells are in the outer and middle EGL but 7% are located in the inner EGL. (**F**) An example of the E14 cerebellar tissue with GFP electroporated granule cells distributed throughout the cerebellar cortex. Some GFP cells in the EGL (**F’**) co-express PH3 (**F”** arrow, insert). Morphologies of cells in the different layers of the EGL can be examined. In the inner EGL cells have bipolar morphologies with long processes yet PH3 positive cells are present among them. Scale bar = 100 μm.

The most comprehensively studied secondary proliferative cell population in the brain is the granule cell precursors (GCPs) within the EGL. An accepted view of EGL assembly, established by Cajal^18^, is that GCPs, which accumulate in this transient secondary epithelium after their birth at, and migration from, the rhombic lip, undergo determined sequential phases of proliferation, morphological elaboration, followed by tangential and radial migration into the inner granular layer (IGL) (Fig. 1A). This three phase sequence is a rational, parsimonious interpretation of the array of morphologies revealed by Golgi staining and is a graphical narrative that has had a profound influence on how morphology and differentiation status of GCPs has been assessed in many species and systems^7–17^, including the chick cerebellum^10^. In this paradigm, proliferating GCPs are localised to the superficial EGL (Fig. 1A, stage a) where they are generally accepted to “have a round soma without any long processes”^15^, although Cajal’s original descriptions identify short, irregular somatic protrusions^18^. Cajal defined a second phase (Fig. 1A, stage b) where post-mitotic cells adopt a bipolar, fusiform and horizontal^15,18^ morphology deep relative to dividing cells of the EGL. These post-mitotic cells exhibit processes of irregular length that can be tipped with growth cones and decorated with spines. These spines are retracted as bipolar cells’ cell bodies transition seamlessly into an inward radial migration towards the IGL, leaving behind the parallel fibres, the cells’ axons (Fig. 1A, stage c). However compelling, does this deterministic and linear interpretation of morphology capture the diversity of GCP behaviour? Given that the neuroblasts of the RMS, for example, retain their ability to divide as they migrate towards the olfactory bulb^19–22^ and express markers characteristic of postmitotic neurons^23–25^, we explored the possible presence of similar developmental features in GCPs in the developing cerebellum.

Given the number of studies of EGL development, there is a surprising dearth of time-lapse imaging of GCP behaviour in an intact EGL, and the few performed do not explore GCP morphology^26,27^. One reason for this is the extraordinary density of cells within the EGL, which exclusively comprises GCPs. This makes the visualisation of detailed morphologies of individual cells difficult. To overcome this, we electroporated a GFP transgene into chick rhombic lip cells in early development and allowed the embryo to grow *in ovo* for ten days. This results in sparse labelling of EGL cells and allowed detailed morphological examination of individual GCPs, which largely correspond to those described previously^10,18^ (Fig. 1B). In fixed tissue and *ex ovo* time-lapse imaging of cerebellar organotypic slices we find that GCPs retain their ability to divide in all layers of the EGL; are highly motile between cell divisions; can elaborate long and complex cellular processes that are retracted prior to cytokinesis; and can express proteins correlated with differentiation, such as TAG1 and NeuroD1, before undergoing final mitoses. Furthermore, we examine the role of NeuroD1, a transcription factor necessary for granule cell differentiation^28–31^, on the development of chick GCPs. Following misexpression of NeuroD1 in early-born GCPs at the rhombic lip, the progenitors differentiate early and fail to populate the EGL. The consequence is a smaller and unfoliated cerebellum with mislocalised cells of abnormal morphologies.

## Results

To observe the morphologies of individual GCPs in the chick EGL we electroporated the rhombic lip progenitors at embryonic day 5 (E5) with a plasmid encoding a GFP transgene flanked by Tol2 sites, alongside a plasmid encoding a Tol2 transposase^32^. This results in a stable genomic GFP expression in a subset of GCPs born at the rhombic lip. We sacrificed embryos at E14 (the peak of GCP proliferation in the chick EGL) and found sparsely labelled rhombic-lip derived cells in the EGL, the molecular layer, and the IGL (Fig. 1B).

We observed cells resembling granule cells at various stages of development that were distributed in a manner consistent with Cajal’s model throughout the cerebellar cortex (Fig. 1A,B). We also see radially migrating postmitotic granule cells as well as a small population of cells in the deeper layers that might represent unipolar brush cells, which are born at the rhombic lip after GCPs (Fig. 1B, arrowheads). To verify the identity of cells within the EGL we stained the tissue for Pax6, a marker of differentiating and mature granule cells (Fig. 1C). We found that all GFP-expressing cells in the inner half of the EGL co-express Pax6, confirming these cells as differentiating, tangentially migrating granule cells (Fig. 1C’). We conclude that all rhombic lip derived, GFP-expressing cells within the EGL are granule cells.

To identify proliferating granule cell precursors among all GFP-expressing granule cells in the EGL we used an antibody against phosphohistone H3 (PH3), a marker of mitosis. PH3 positive cells were found in all layers of the EGL, including the inner EGL (Fig. 1D). We mapped the distribution of PH3 positive cells, by dividing the EGL into three equal sectors (outer, middle, inner EGL, Fig. 1D’). The highest proportion of PH3 positive cells was found in the outer EGL (49%), followed by the middle EGL (44%) with a minority of cells located within the inner EGL (7%) (Fig. 1E). We assessed the complexity of PH3-positive cells and found that the morphology of dividing cells often reflects their location within the EGL (Fig. 1F). This suggests that dividing granule cells can display an unexpected diversity of form.

**Figure 2:**
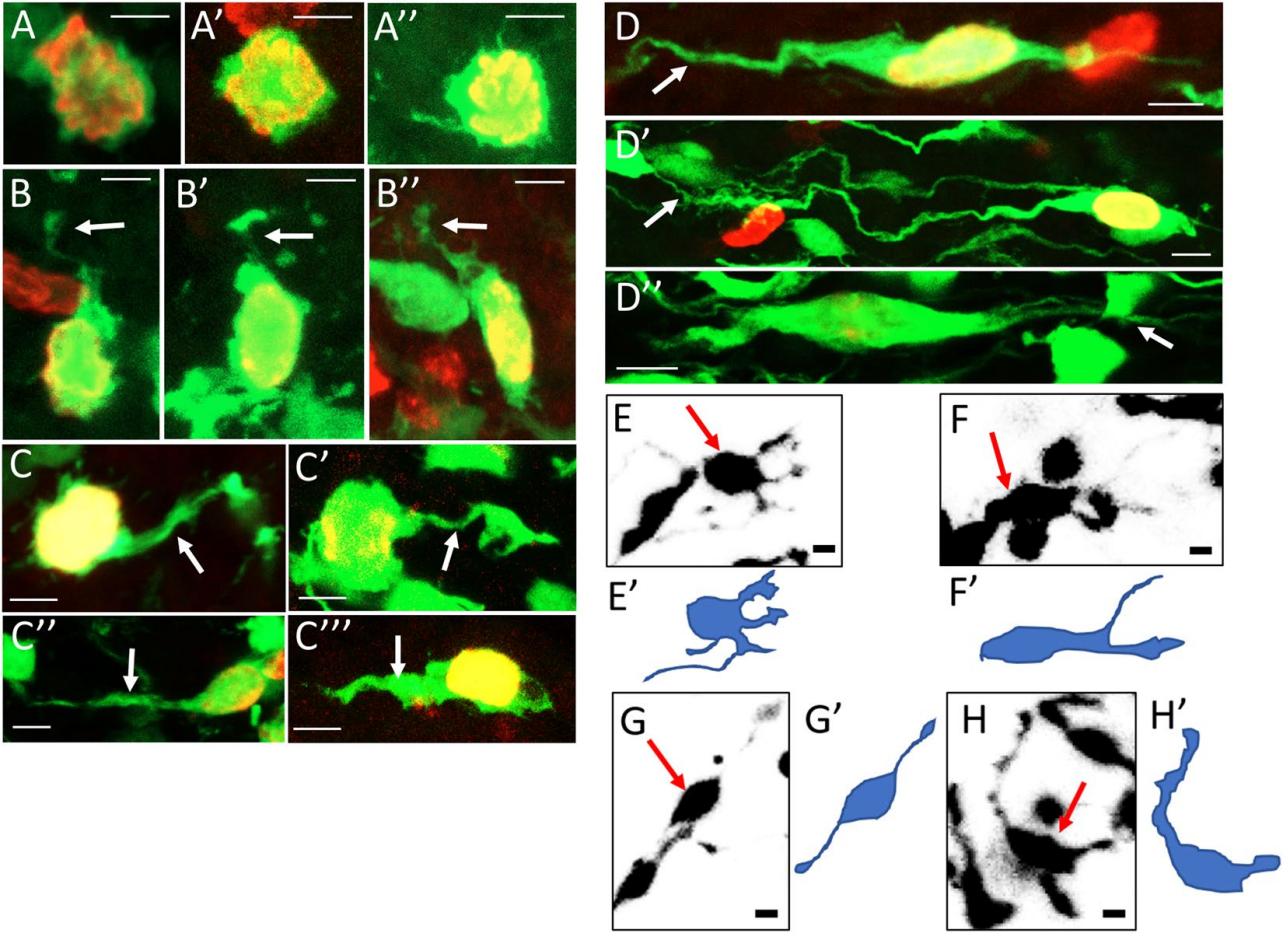
Granule cell precursors have complex morphologies and can extend processes. High magnification composite images of individual GFP (green) labelled cells that co-express PH3 (red). (**A**) Cells that have a rounded morphology. (**B**) Cells that appear to have a basal attachment. (**C**,**D**) Cells with a variety of cellular processes (white arrows), some resembling leading processes. (**E**–**H**) One Z-layer stills of four different GCPs captured during time-lapse imaging of cerebellar organotypic slices. The cells (red arrows) extended different types of processes before mitosis. (**E’**–**H’**) An outline of the cell based on multiple Z-layers collected is presented for better visualisation of the morphologies. Scale bar = 5 μm.

Mitotic cells located in the outer EGL are mostly spherical and lack long cellular processes. They can however extend short lateral protrusions (Fig. 2A) or basal attachments which protrude directly to the pial surface (Fig. 2B). PH3 positive cells located in deeper sectors, within the middle and inner EGL, can possess long and elaborate cellular processes (Fig. 2C,D). These can have claw-like extensions (Fig. 2C-C’) or more elongated processes (Fig. 2C”-C”’). While the overall geometry of dividing cells becomes increasingly bipolar in the inner EGL (Fig. 2D), there may still be multiple filamentous processes at either pole of the cell (Fig. 2D”). Cells with long, extended processes (<40 µm in length), and horizontally elongated nuclei (Fig. 2D) share the characteristics of tangentially migrating neurons. Time-lapse confocal imaging of live GCPs within these deeper layers imaged in organotypic slice culture *ex-ovo*, revealed that processes continuously change their shape, length and direction of extension (Fig. 2E-H, Supplementary Movie S1). We therefore conclude that a proportion of mitotically active GCPs can be located in the middle and inner EGL and can extend elaborate cellular processes before dividing.

**Figure 3:**
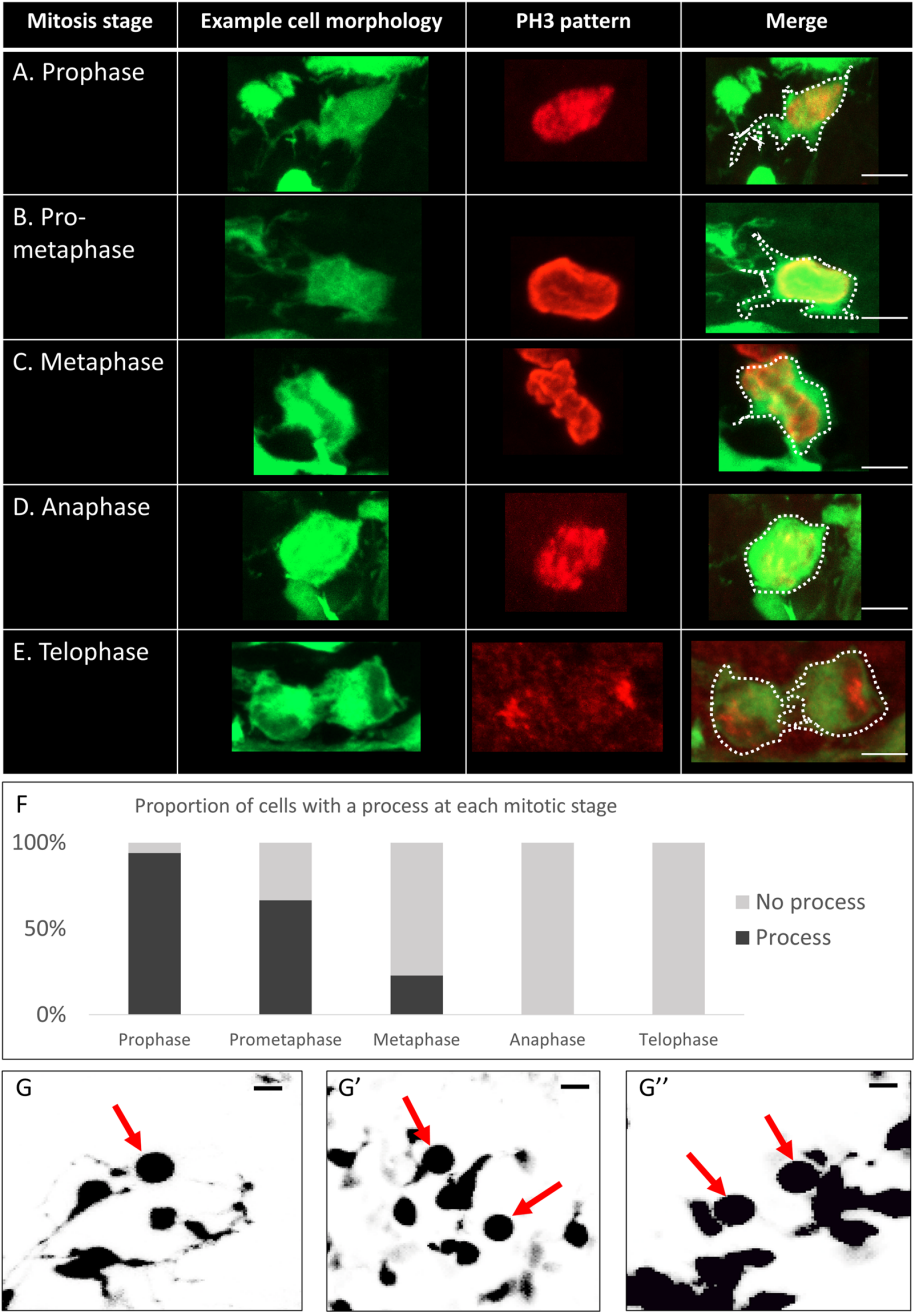
Granule cell precursors retract all processes before cell division. (**A**–**E**) Examples of typical cellular morphologies of GCPs at different stages of mitosis, characterised by distinctive PH3 condensation pattern (red). White dots delineate the cell morphology on merged images. (**A**) A cell in prophase with an example of a short, extended process. Prophase cells typically have even longer processes. (**B**) A cell in pro-metaphase with processes that appear to be retracting. (**C**) A cell in metaphase with very short processes. (**D**) A cell in anaphase with no processes visible. (**E**) A cell in late telophase. There are no processes extended at this point. (**F**) Proportions of cells with an extended process at different stages of mitosis. As the mitotic cell approaches anaphase and telophase, it is increasingly less likely to be extending a process (n = 154 cells from 3 cerebella). (**G**) Stills of five different GCPs captured minutes prior to cytokinesis during time-lapse imaging of cerebellar organotypic slices. The cells (red arrows) have no processes and a completely round morphology. All observed cells in time-lapse imaging underwent this stage of morphological transition before division (n = 16). Scale bar = 5 μm.

Because PH3 defines a relatively narrow M-phase window in mitosis, the rapid remodelling of processes makes it possible that even very long cellular extensions are retracted during cytokinesis. The number of cell division events recorded by time-lapse imaging in the outer EGL were too few to answer this question. We therefore returned to PH3 label and used condensation patterns of DNA at high magnification to characterise the morphologies of GCPs at different stages of mitosis (Fig. 3). We found that the proportion of GCPs that extend processes decreases as the cells pass through prophase (Fig. 3A), pro-metaphase (Fig. 3B) to metaphase (Fig. 3C). During anaphase (Fig. 3D) and telophase (Fig. 3E), when cells undergo cytokinesis, processes are completely withdrawn. Quantification in Fig. 3F shows that nearly all cells observed in prophase (96%) extended some form of a process, whereas very few cells in metaphase (23%) and no cells in anaphase or telophase extended processes. These observations were supported by time-lapse movies, which showed dividing cells in the EGL consistently retracting their cellular processes and completely rounding up before division (Fig. 3G, Supplementary Movie S2).

**Figure 4:**
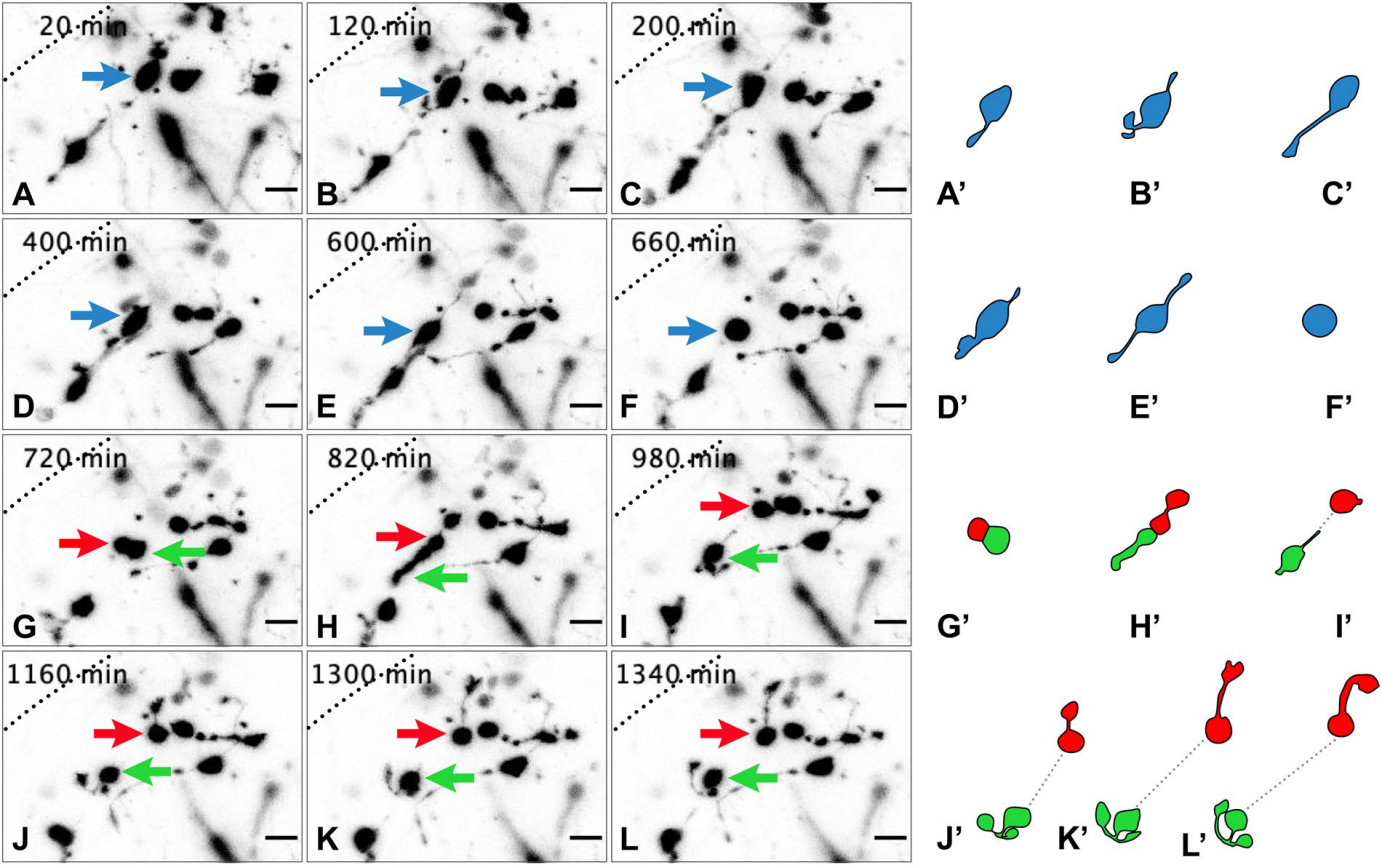
Time-lapse movies of dividing granule cell precursors show highly dynamic morphological transitions. A selection of chronological frames from time-lapse imaging of a cerebellar slice. A dividing GCP (blue arrows A–F; blue cells A’–F’) and the behaviour of two daughter cells (red and green arrows G-L; red and green cells G’-L’) were observed over 22 hours at 20-minute intervals. Processes can be seen extending from the cell bodies (A–L). Traces of each cell of interest based on all Z-layers (A’–L’) are provided for easier visualisation. (**A**) At the first time point (20 min) the mother cell (blue arrow) is extending one leading process. Whether it has a trailing process cannot be determined due to obstruction from another cell. (**B**) Within 2 hours, the cell has migrated a small distance in a medio-lateral direction and extends two small, thin processes. The leading process has bifurcated and a growth cone is visible at the tip of one of the processes. (**C**) At 200 min, the cell continues to extend its leading process, which has become longer and thinner. (**D**) At 400 min, the cell has migrated further and extends a thicker and shorter process. (**E**) Within 10 hours the cell once again shows two long, thin processes from both sides prior to (**F**) the cell retracting all processes and rounding up for cell division. (**G**) Within an hour of the parallel division, the two daughter cells begin to separate (red and green arrows; G, red and green cells; G’). (**H**) Both cells extend their own processes in opposite directions. One daughter cell extends a process with a very broad growth cone (red), whereas the other daughter cell has a longer and much thicker process (green). (**I**) The daughter cells separate from each other as each migrates in a different direction. (**J**–**L)** One of the daughter cells (green arrow J-L; green cell J’- L’) extends two processes that bifurcate into a T-shaped process, resembling the formation of a rudimentary parallel fibre. The other daughter cell (red arrow J-L; red cell J’- L’) laterally extends a longer process that has a large, very motile growth cone. At the end of the time-lapse (22 hours), the cell (red arrow L; red cell L’) has directed its process back towards the inner EGL. Movie available as Supplemental Movie S3. Pial surface: dotted black line. Scale bar = 20 μm.

An example of a bipolar cell undergoing division is shown in Fig. 4, (Supplementary Movie S3). Here, 12 frames spanning 26 hours show the division of a bipolar GCP in the middle EGL. The GCP extends leading- and trailing- like processes before mitosis and its cell body migrates a short distance (~10 µm), which is followed by retraction of all processes, the cell rounding up and dividing parallel to the pial surface. Following mitosis, the two daughter cells migrate in opposite directions from one another and extend their own processes, one forming a rudimentary T-shaped cell process reminiscent of forming parallel fibres. This type of behaviour was observed in the majority of dividing GCPs imaged, whereby following division, the daughter cells extended leading processes in opposite directions and migrate away from each other (data not shown). Cells were not imaged sufficiently long to follow the fate of the daughter cells. Most divisions observed in the time-lapse movies occurred perpendicular to the pial surface (64%) with the rest being parallel (36%).

**Figure 5:**
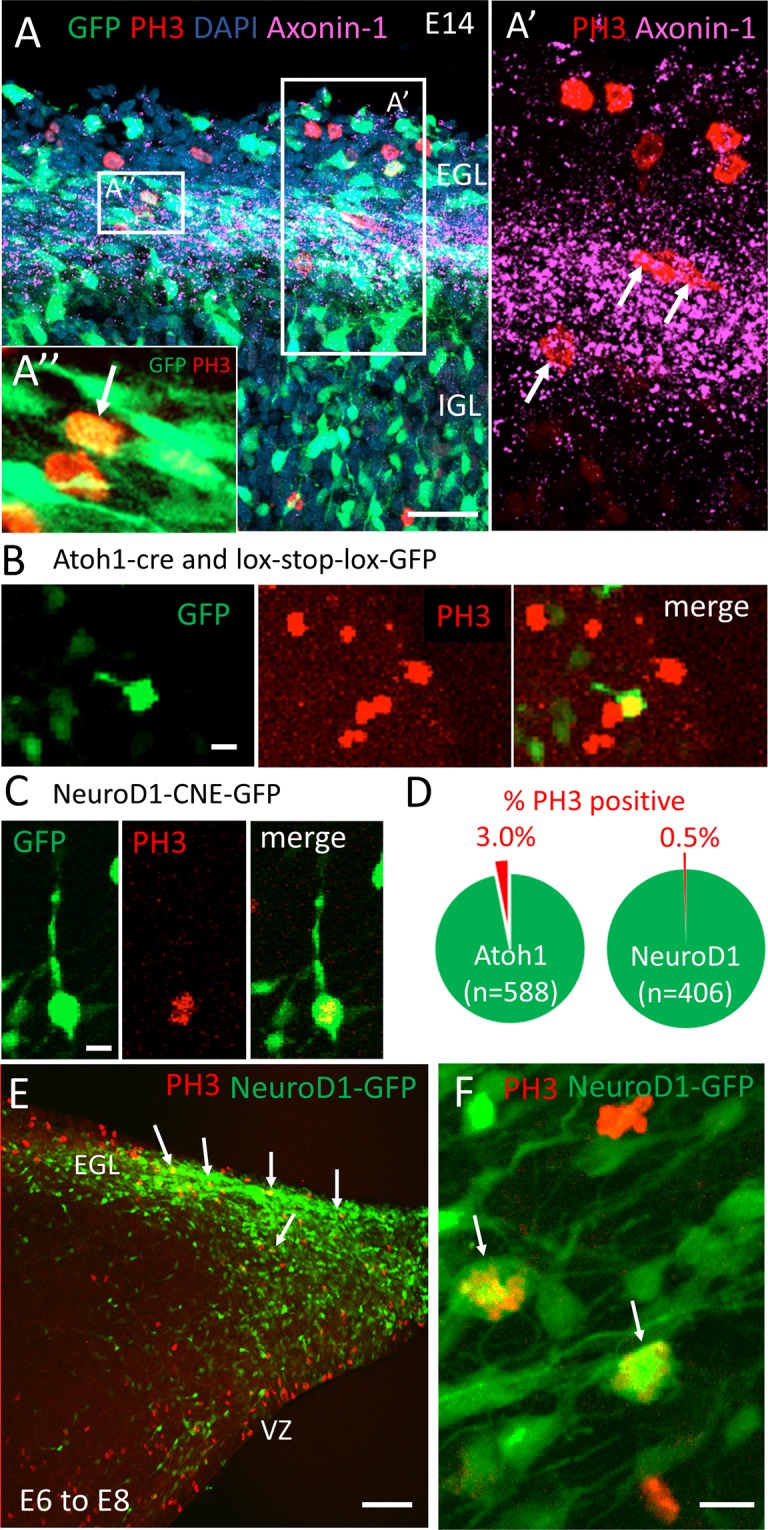
Mitotically active granule cell precursors can express proteins associated with differentiation such as TAG-1 (Axonin-1) and NeuroD1. (**A**) Cerebellar tissue from chick embryos electroporated with Tol2:GFP at E4 and fixed at E14 was immunostained for PH3 (red) and TAG1/Axonin-1 (magenta). DAPI shown in blue. (**A’**) Co-localization of mitotic, PH3 expressing cells and Axonin1 in the inner EGL. (**A”**) A GFP expressing cell expressing PH3 is surrounded by bipolar cells with long processes in the inner half of the EGL. (**B**,**C**) E14 cerebellar slices were electroporated with Atoh1-cre and lox-stop-lox-GFP plasmids (**B**) or NeuroD1-CNE-GFP plasmid (**C**) and stained for PH3. (**D**) Co-localization of PH3 and GFP was observed in 3% of cells where GFP was driven by the Atoh1 enhancer, and in 0.5% of cells where GFP was driven by NeuroD1 enhancer element. (**E**) A plasmid encoding full length NeuroD1-GFP driven by β-actin promoter was electroporated into the rhombic lip of E6 embryos, fixed two days later, at E8, and stained for PH3. A sagittal cut through the cerebellum reveals cells co-expressing NeuroD1-GFP and PH3 in the forming EGL (arrows). VZ = ventricular zone. (**F**) A higher magnification of the EGL cells co-expressing NeuroD1-GFP and PH3 (arrows). Scale bar = A,E = 50 μm, B,C,F = 10 μm.

The elaborate processes found on a subset of proliferative GCPs raised the question of whether the cells have initiated a differentiation pathway at the time of cell division. Previous studies have suggested that Axonin1 (TAG1 in mouse), which is important for axon pathfinding in the EGL^33^ and marks initial differentiation of granule cells, can co-label alongside proliferative markers^34,35^. Consistent with this, Axonin1 expression in the chick cerebellum characterises a broad domain in the inner half of the EGL that overlaps with some PH3 positive cells (Fig. 5A), suggesting that some progenitor cells may express markers of differentiating neurons prior to final mitoses. Another transcription factor associated with the onset of differentiation of granule cells is NeuroD1^31,36–39^. Due to the absence of an antibody in chick for NeuroD1 we used a previously described NeuroD1 expression reporter (NeuroD1-CNE-GFP)^30^. We assessed the number of PH3 positive EGL cells that co-label with GFP following electroporation of the NeuroD1-CNE-GFP plasmid into E14 cerebellar slices^40^ (Fig. 5B). 2 out of 406 GFP-positive cells were co-labelled with PH3, compared to 19 out of 588 cells electroporated when GFP was driven by an Atoh1-cre plasmid that reports activity of proliferating granule cell precursors (Fig. 5C,D). This suggests that it is possible for PH3 positive granule cell precursors to have high levels of NeuroD1 gene activation and that NeuroD1 might be expressed in ≈15% of mitotic GCPs.

Our results show that cells that have initiated a differentiation pathway may retain proliferative potential. To test this hypothesis, we drove differentiation in a larger pool of GCPs by electroporating a full length NeuroD1-GFP plasmid into GCPs at the rhombic lip at E6, when first GCPs are born. NeuroD1 is considered a marker of postmitotic granule cells^31,36–39^. We previously showed that misexpression of NeuroD1 at E4 has the capacity to block EGL assembly^30^. Interestingly, following misexpression at E6, many GCPs within the forming EGL at E8 misexpressed exogenous NeuroD1-GFP and yet co-expressed PH3, suggesting that progenitor cells misexpressing a differentiation factor can nonetheless undergo mitosis (Fig. 5E,F).

**Figure 6:**
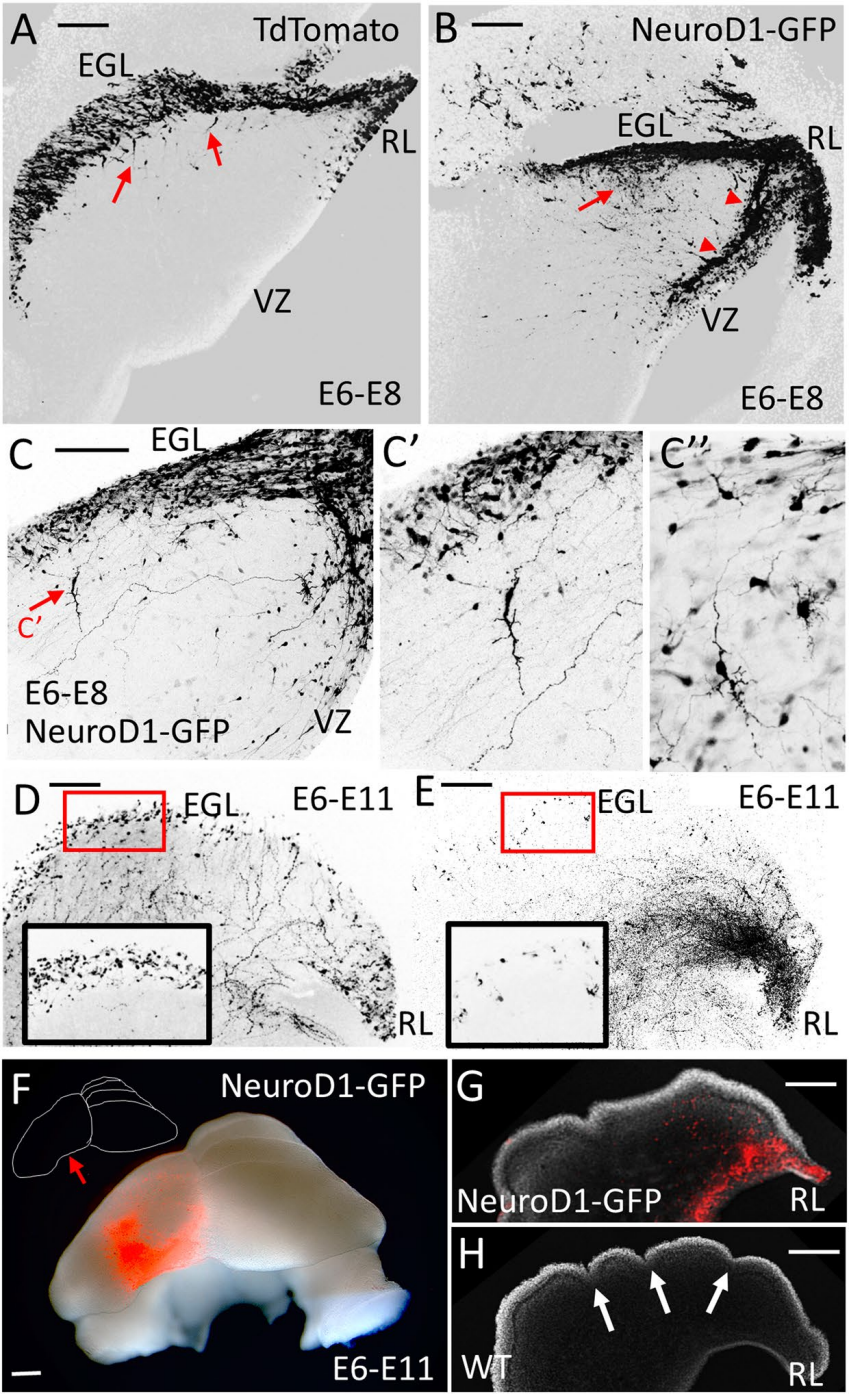
Misexpression of NeuroD1 results in defects in granule cell migration and proliferation and affects the overall growth and foliation of the cerebellum. (**A**–**C**) Rhombic lips of E6 embryos were electroporated with a control TdTomato plasmid (**A**) or full-length NeuroD1-GFP (**B**,**C**), fixed and cut sagittaly into 150 μm sections at E8. (**A**) In control electroporation, most electroporated cells populate the forming EGL, with a few cells starting to migrate radially from the EGL into the IGL (red arrows). (**B**) When NeuroD1 is misexpressed, EGL formation still occurs, but a large number of cells migrate into the IGL (red arrow) and exhibit morphologies reminiscent of differentiated granule cells. Additionally, a large stream of cells migrates ventrally from the rhombic lip (RL) towards the ventricular zone (VZ) (red arrowheads). (**C**) Cells misexpressing NeuroD1 occupy abnormal positions within the cerebellar anlage and show a variety of morphologies, some of which resemble differentiating granule cells. (**D**) At E11, following a control TdTomato electroporation at E6, a thick EGL formation is observed (box in D, insert) as well as a scattering of post-mitotic granule cells and parallel fibres deep to the surface, in a molecular layer that has been sectioned obliquely. (**E**) At E11, following a NeuroD1-GFP misexpression, the EGL is nearly devoid of cells (box in E, insert), which are instead located close the rhombic lip (RL), in deep tissue layers. (**F**) An example of whole cerebellum electroporated at E6 with NeuroD1-GFP on one side of the rhombic lip only (red, arrow); anterior side facing up. The electroporated side of the cerebellum shows reduced size and lack of foliation pattern seen on the unelectroporated side (see the insert drawing). (**G**,**H**) A sagittal section through the cerebellum shown in F showing differences in the foliation pattern between NeuroD1-GFP electroporated side (**G**) and the unelectroporated side (**H**). Arrows point towards the developing folia on the wild type (WT) side. Scale bar = A–C = 200μm, D–H = 500 μm.

We confirmed that cells expressing ectopic NeuroD1 are indeed driven to early differentiation by examining the distribution of electroporated cells two and five days after an E6 electroporation. At E8, after electroporation of control tdTomato plasmid into the rhombic lip, the EGL of control embryos is populated with fluorescent migratory derivatives (Fig. 6A). Following misexpression of NeuroD1, however, there is both a limited migration of rhombic lip derivatives into the EGL and their ectopic distribution at the boundary between the mantle layer and the ventricular zone (Fig. 6B). While there is almost no radial migration from the EGL into the IGL of control postmitotic granule cells at E8 (Fig. 6A), misexpression of NeuroD1 induces mature granule cell morphologies prematurely (Fig. 6C). At later stages (E11), the consequence of NeuroD1 misexpression, when compared to a normal EGL (Fig. 6D and inset), is a depletion of GFP labelled GCP precursors in the EGL as well as mislocalization of rhombic lip derived cells (Fig. 6E and inset). In a unilateral electroporation this results in a smaller cerebellum ipsilateral to NeuroD1 misexpression (Fig. 6F) and is reflected in a loss of foliation at the electroporated side, (Fig. 6G) compared to the normal foliation in the non-electroporated side of the E11 cerebellum (Fig. 6H).

## Discussion

In this study, we demonstrate in chick that GCPs divide in all layers of the EGL; can extend elaborate processes between cell divisions; retract all processes prior to cytokinesis; are able to undergo short migration movements before mitosis; and can express differentiation-associated genes whilst mitotically active. Additionally, GCPs can divide perpendicular or parallel to the pial surface and the daughter cells tend to migrate away from one another in the medio-lateral direction. These results raise questions about the nature of the relevance of inner and outer EGL designations for proliferative versus non-proliferative GCPs and whether transition through the inner EGL represents a deterministic, or a one-way, step towards radial migration.

The widespread distribution of mitotic cells through the depth of the EGL poses questions about the factors that regulate the overall amplification of the population. Recent evidence of a stem cell population in mice that is capable of repopulating the EGL in response to injury^41,42^ suggests a far less determined developmental path to a granule cell than previously imagined. It seems unlikely that any GFP labelled cells in this study arose from this deeply situated population of cells. However, it is possible that the depletion of the EGL following NeuroD1 over expression is partly compensated for by an as yet uncharacterised population of equivalent cells in birds.

In common with some intermediate progenitors in the cortex, GCPs appear to extend a basal attachment, which has been proposed to be essential for GCP proliferation^11,43,44^. Our data suggest that this basal attachment is not a common feature of all dividing cells but rather is a feature limited to some cells within the outer EGL. One possibility is that the attachment to the basal lamina is linked to the expression of Atoh1. This transcription factor is essential for transit amplification and symmetrical proliferative divisions of GCPs^45^ and is linked to the ability of cells to occupy a subpial membrane^46^. As GCPs leave the outer EGL and switch off Atoh1 expression, they might lose their proliferative capacity and undergo asymmetrical or terminal neurogenic divisions only. One question that remained beyond our technical capacity was to determine cell fate of the electroporated cells by imaging multiple rounds of division within the EGL.

**Figure 7:**
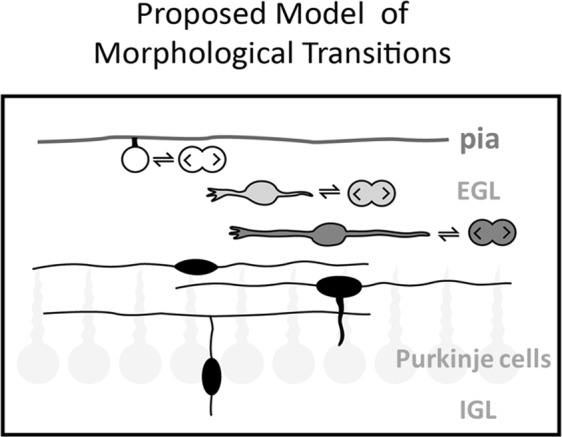
Proposed model of GCP differentiation in the EGL. The current model of GCP morphological transitions postulates that mitotically active, polyhedral or round cells, with a possible basal attachment to the pial surface proliferate in the outer layer of the EGL. Only after the cell becomes postmitotic, it extends small horizontal processes and begins tangential migration. Those processes continuously extend medio-laterally until the cell makes a switch to radial migration, at which point the horizontal processes are considered nascent parallel fibres. We suggest that this model is incomplete. The proposed model retains and confirms the morphological features of GCs in the EGL, but suggest that cells previously considered postmitotic, can in fact be proliferative. Divisions of GCPs occur in all layers of the EGL and cells with long horizontal processes are able to undergo mitosis by retracting their processes and rounding up before division. In the proposed model, white shading represents a highly proliferative precursor, and black shading denotes a postmitotic cell. As the cell migrates through the EGL and is exposed to diverse signalling, its proliferative potential declines. Consequently, the number of times the cell divides decreases as the cell transitions through EGL layers, with many mitoses in the outer EGL and very occasional mitoses in the inner EGL.

Rhombic lip-derived cerebellar GCPs are conventionally understood to undertake a stereotyped sequence of morphological steps as they transition through the layers of the developing cerebellum. Our results show that the sequence of morphological differentiation of GCPs does not fully follow the linear sequence anticipated from Golgi studies^18^. Rather, our data suggests that GCPs lose their proliferative potential as they migrate through the layers of the EGL, as depicted in the model in Fig. 7. This is experimentally supported by misexpression of NeuroD1, which, as shown elsewhere^29^, results in a smaller cerebellum but does not abolish proliferation within multipolar granule cells.

Other studies have shown that mitotic granule cell precursors can express a number of markers associated with postmitotic cells such as Axonin1/TAG1^35^ and NeuroD1^47^, as well as p27^34^, supporting a model whereby increasing the expression of proteins associated with differentiation encourages cells towards a terminal division, but does not cease mitotic activity per se. It remains to be shown whether these types of morphological changes occur in the mammalian cerebellar system. Currently, no rodent studies have explored the morphological features of individual GCPs *in vivo*. It is possible that the morphological plasticity that we observe in chick GCPs is a general feature of GCP differentiation in all amniote species.

Interestingly, both the morphological transitions and the gradual transcriptional changes of GCPs resemble those of the neuroblasts migrating within the RMS^19–22^. RMS neuroblasts go through repeated stages of migration, process retraction, and division on route to the olfactory bulb^19^. They also have a transcriptome that gradually shifts from a program controlling proliferation to one that modulates migration and differentiation^4^. While migrating in the RMS, neuroblasts receive a plethora of stimuli that modify transcription according to the local microenvironment. It would be interesting to examine whether GCPs, which migrate tangentially within the EGL for shorter distances^15^, also respond to local stimuli to guide their development and what the identity of such stimuli might be. The response of stem cells in the white matter that can respond to cues in the EGL^41,42^ support this hypothesis. It seems likely that local cues that drive stem cell repopulation of a damaged cerebellar might also guide rhombic-lip derived granule cells to populate the cerebellum appropriately during normal development.

## Methods

### Animals

All experiments on chicken embryos were carried out in accordance with the relevant Home Office guidelines and regulations.

### Plasmids

pT2K-CAGGS-EGFP and pCAGGS-T2TP were described previously^48^ and are referred to as Tol2:GFP in this study. pT2K-CAGGS-EGFP is a Tol2 transposon-flanked EGFP and pCAGGS-T2TP codes for Tol2 transposase. Upon co-electroporation of pT2K-CAGGS-EGFP and pCAGGS-T2TP plasmids, the resulting transposon construct is excised from the plasmid and integrated into the host genome in proliferating precursors. NeuroD1-CNE-GFP plasmid has been reported previously^30^. In short, a conserved non-coding element drives the expression of GFP that recapitulates endogenous NeuroD1 expression. A CAGGS-TdTomato plasmid was used as a control electroporation plasmid. NeuroD1-GFP misexpression construct has been described previously and was cloned using full length *NeuroD1* sequence following a β-actin promoter^30^. IRES sequence was inserted between the *NeuroD1* sequence and the EGFP. The Atoh-cre plasmid has been reported previously^49^; we replaced the *LacZ* sequence with *Cre-recombinase* sequence to conditionally report expression of Atoh1 when combined with lox-stop-lox-GFP plasmid.

### *In ovo* electroporation

Fertilised chicken eggs were incubated at 38 °C for 4–6 days. The eggs were drained a day before electroporation with a hypodermic needle; 3–4 ml of egg albumen was removed. The eggs were then windowed using egg scissors and the embryo was located and its position manipulated for ease of access. DNA constructs at 1–2 µg/µl were mixed with trace amounts of fast green dye (Sigma) and injected into the fourth ventricle directly below the rhombic lip using a glass needle. Where two or more constructs were co-electroporated both were mixed in equal amounts, each at concentration of at 1–2 µg/µl. The negative electrode was placed underneath the embryo at the level of the rhombic lip and the positive electrode was placed on top of the embryo at the same level. The cerebellar rhombic lip was targeted this way. Three 50 ms/10 V square waveform electrical pulses were passed between the electrodes so that DNA entered the right side of the neural tube. Eggs were then treated with Tyrodes solution (1 ml) and sealed back. Eggs were incubated at 38 °C until the embryos were harvested at appropriate experimental age (E8-E14). Dissected hindbrains (E8-E10) or cerebella (E11-E14) were fixed overnight at 4 °C in 4% PFA in PBS and then processed as needed for histology, mounting and imaging. All procedures were carried out with Home Office approval and were subject to local Ethical Committee review at King’s College London.

### Live imaging of cerebellar slices

The rhombic lip was electroporated with Tol2:GFP *in ovo* at E4 as described above. The cerebella were dissected at E14 in cold HBSS and processed into 250 µm sagittal slices using a tissue chopper. Tissue with a good representation of fluorescently labelled cells (identified using an epifluorescent stereomicroscope) was selected for time-lapse filming. Slices were transferred using a plastic pipette into a pre-assembled coverslip with a glass ring attached with silicon grease to create vacuum in the Rose chamber. The chamber is constructed from two 25 mm^2^ coverslips, a silicon spacer, a metal planchet milled to accept a condenser lens. The whole assembly is held together by two metal clips attached to the sides. The chamber can be filled and drained using two 25 G needles and a syringe. Excess liquid transferred with the tissue was removed from the cover slip, making sure that the cerebellar slice lies flat on the cover slip. 500 µl of rat tail collagen prepared to a neutral pH was then added on top of the slice in the glass ring, making sure that the slice remains close to the surface of the cover slip. The cover slip with the cerebellar slice was then incubated at 37 °C 5%CO_2_ for 30 min to 1 hr. After the collagen has set, another cover slip was placed on top of the ring and the rest of the Rose chamber was assembled. 2 ml of pre-incubated (37 °C 5%CO_2_) culture medium was added to the sealed chamber and the preparation was immediately imaged using a Nikon Eclipse EZ-C1 confocal microscope with a 20x objective lens overnight (12–28 hrs) with 20 min intervals between time points. For analysis, variable z-stack projections were chosen from the whole z- stack, depending on the best combination to observe specific cell behaviours.

### Cerebellar slice electroporation

Fertilised eggs were incubated at 38 °C for 14 days. The chicken embryo was decapitated at E14 and the cerebellum was dissected out in ice cold HBSS. The cerebellum was sectioned using a tissue chopper in a sagittal orientation into 300 µm thick slices. The sliced cerebellum was covered in ice cold HBSS and transferred into a 60 mm Petri dish containing ice cold fresh HBSS. Under a dissecting microscope illuminated with a fibre optic light source, individual slices were separated using watchmaker forceps. Slices to be electroporated were selected based upon their tissue integrity and medio-lateral position.

An electroporation chamber was constructed by fixing the anode of an electroporator to the base of a 60 mm Petri dish with insulation tape. Approximately 1 ml of HBSS was added to cover the electrode. A 0.4 µm culture insert was placed on top of the electrode covered in HBSS. Identified slices (up to five per culture insert) were transferred onto culture insert. The slices were separated and allowed to settle onto culture insert in a sagittal orientation. Excess HBSS was removed. 5 μL DNA solution (at a concentration of 1 μg/μL) diluted with 20% fast green was pipetted over the surface of targeted region of a slice. The cathode was placed over desired targeted tissue, which was electroporated with 3 × 10 V, 10 ms duration pulses. The surface tension of the liquid was used to maintain conductance. DNA delivery and electroporation to multiple regions of EGL was repeated on each individual cerebellar slice as desired. Upon completion of electroporation, culture insert was transferred to 30 mm Petri dish with 1 ml of pre-warmed culture medium. Cultures were incubated at 37 °C/6% CO_2_ for two days. Culture medium was replaced every 24 hours with fresh pre-warmed medium. Following culture, slices were fixed on culture inserts for 1 hour at room temperature in 4% paraformaldehyde (or overnight at 4 °C). Slices were processed for immunohistochemistry as described previously^40^. Fluorescent confocal images were taken with Zeiss LSM 800 microscope or Olympus FV 500. Z-stack projections were compiled using ImageJ. Z-stacks were taken at 1–20 µm intervals.

### Histology and immunohistochemistry

Electroporated E14 chicken cerebellum was fixed in 4% PFA overnight at 4 °C. The tissue was then washed in PBS 3 times for 15 min and then transferred into 10% sucrose (Sigma) in PBS for 30 min, then 20% sucrose solution for 30 min and finally 30% sucrose solution overnight. The tissue was then transferred into OCT compound (VWR) in moulds and placed on dry ice or liquid nitrogen to freeze. The tissue was then stored at −80 °C overnight. For sectioning, the blocks were placed at −20 °C an hour before sectioning. The blocks were cut and mounted on cryostat chucks using OCT compound and sectioned using a Zeiss Microm HM 560 cryostat at 50 µm thickness and transferred onto Superfrost Plus slides (VWR). The sections were allowed to air dry for two hours and were stored at −80 °C long term and −20 °C short term. Cryostat sections were defrosted for at least 30 min at room temperature before immunohistochemistry. The slides were then washed three times for 5 min in PBS. Slides were then covered in 500–800 µl block (1% normal goat serum, 0.2% Triton x100 in PBS) and incubated for 30 min at RT. Primary antibody was diluted at an appropriate concentration in the blocking solution. After the blocking solution was removed from the slides, 150–200 µl of the antibody solution was added onto the slide and covered with parafilm to prevent drying out. Incubation was performed overnight at 4 °C. The next day, primary antibody was washed off with PBS three times for 5 mins. Secondary antibody was diluted in block solution and put onto the slides for 2 hrs at RT. The slides were then washed with PBS three time for 5 mins and covered with a coverslip using Fluoroshield mounting medium with DAPI (Abcam). Fluorescent confocal images of all fixed tissues were taken with Zeiss LSM 800 microscope or Olympus FV 500. Z-stack projections were compiled using ImageJ. Z-stacks were taken at 1–20 µm intervals.

The primary antibodies used in this study include calbindin (rabbit, SWANT 1:2000), Phosphohistone H3 (rabbit, Cell Signalling, 1:150), GFP (mouse, Abcam, 1:1000), GFP (rabbit, Life Technologies, 1:1000), Axonin1 (mouse, Hybridoma Bank, 1:100). Appropriate secondary antibodies: Alexa Fluor 488, 568, 633 (Thermo Fisher Scientific, 1:500).

## Supplementary information

Supplementary Movie 1

Supplementary Movie 2

Supplementary Movie 3

Supplementary information

## Data Availability

The data generated and analysed during the current study are available from the corresponding author on reasonable request.
