# Supplementary material for: Mitotic granule cell precursors undergo highly dynamic morphological transitions throughout the external germinal layer of the chick cerebellum
Source: Sci Rep. 2019 Oct 23;9:15218. doi: 10.1038/s41598-019-51532-y (PMC6811643; doi:10.1038/s41598-019-51532-y)
Supplement: Supplementary file 4 — Supplementary information [file 41598_2019_51532_MOESM4_ESM.pdf]

**Supplementary data**

# Mitotic granule cell precursors undergo highly dynamic morphological transitions throughout the external germinal layer of the chick cerebellum

Michalina Hanzel[1], Victoria Rook[1,2], Richard JT Wingate[1]*

*Corresponding author (richard.wingate@kcl.ac.uk)

[1] MRC Centre for Neurodevelopmental Disorders
King's College London, Institute of Psychiatry, Psychology and Neuroscience
4th floor New Hunt's House, Guy's Campus
London
UK

[2] School of Biological and Chemical Sciences,
Queen Mary University of London,
Mile End Road
London, E4 1NS
UK
Mitotic granule cell precursors undergo highly dynamic morphological transitions throughout the external germinal layer of the chick cerebellum

Michalina Hanzel[1], Victoria Rook[1,2], Richard JT Wingate[1]*

*Corresponding author (richard.wingate@kcl.ac.uk)

[1] MRC Centre for Neurodevelopmental Disorders
King's College London, Institute of Psychiatry, Psychology and Neuroscience
4th floor New Hunt's House, Guy's Campus
London
UK

[2] School of Biological and Chemical Sciences,
Queen Mary University of London,
Mile End Road
London, E4 1NS
UK

**Supplementary Movie 1**

A time-lapse movie showing a dividing GCP extending and retracting multiple, highly motile processes before cell division.

**Supplementary Movie 2**

A time-lapse movie showing two dividing GCPs retracting all processes and rounding up before cell division.

**Supplementary Movie 3**

A time-lapse movie showing a dividing GCP described in Figure 5.
